# Sensitivity and Specificity of Examination Maneuvers for Carpal Tunnel Syndrome: A Meta-Analysis

**DOI:** 10.7759/cureus.42383

**Published:** 2023-07-24

**Authors:** Yagiz Ozdag, Yirui Hu, Daniel S Hayes, Shahid Manzar, Anil Akoon, Joel C Klena, Louis C Grandizio

**Affiliations:** 1 Orthopaedic Surgery, Geisinger Commonwealth School of Medicine, Danville, USA; 2 Epidemiology and Health Administration, Geisinger Commonwealth School of Medicine, Danville, USA

**Keywords:** specificity, sensitivity, physical examination, hand surgery, meta-analysis, carpal tunnel syndrome

## Abstract

Our purpose was to assess the diagnostic validity (sensitivity (Sn) and specificity (Sp)) of physical examination maneuvers for carpal tunnel syndrome (CTS). This meta-analysis utilized the Preferred Reporting Items for Systematic Reviews and Meta-Analyses (PRISMA) checklist. Studies assessing exam maneuvers (including components of the CTS-6) for CTS were identified in MEDLINE (Medical Literature Analysis and Retrieval System Online) and Embase (Excerpta Medica Database) databases. Assessed maneuvers assessed included: Phalen's test, Tinel's sign, Durkan test, scratch-collapse test, Semmes-Weinstein monofilament (SWM), and static 2-point discrimination (2PD) test. Data extracted included: article name, total number of subjects/hands, type of exam, and exam Sn/Sp. Forest plots were presented to display the estimated Sn/Sp and boxplots were used to demonstrate the locality, spread, and skewness of the Sn/Sp through the quartiles. After screening 570 articles, 67 articles involving 8924 hands were included. Forty-eight articles assessed Phalen's test, 45 assessed Tinel's sign, 21 assessed the Durkan test, seven assessed the scratch-collapse test, 11 assessed SWM, and six assessed the static 2PD test. Phalen's test demonstrated the greatest median Sn (0.70, (Q1, Q3): (0.51, 0.85)), followed by the Durkan test (0.67, (Q1, Q3): (0.46, 0.82)). 2PD demonstrated the highest median Sp (0.90, (Q1, Q3): (0.88, 0.90)), followed by SWM (0.85, (Q1, Q3): (0.51, 0.89)). There is considerable variability with respect to the validity of physical exam tests used in the diagnosis of CTS. Upper-extremity surgeons should be aware of inherent limitations for individual exam maneuvers. In the absence of a uniformly accepted diagnostic gold standard, a combination of exams, along with pertinent patient history, should guide the diagnosis of CTS.

## Introduction and background

Carpal tunnel syndrome (CTS) is frequently encountered in primary care and subspecialty clinics and remains the most common form of peripheral compressive neuropathy in the upper extremity [1-3]. A combination of an accurate patient history and a focused physical examination is required for the diagnosis of this clinical condition. The American Association of Orthopaedic Surgeons (AAOS) recently modified the clinical practice guidelines (CPGs) for CTS where electrodiagnostic studies (EDS) are no longer required for cases without diagnostic uncertainty [4]. However, the routine utilization of EDS in uncomplicated CTS remains controversial. Previous reports have indicated that 26% of hand surgeons require EDS prior to consultations relating to CTS [5]. The use of EDS has been shown to increase healthcare costs and does not change the likelihood of diagnosing CTS in cases without diagnostic uncertainty [6,7].

For this reason, the CTS-6 was developed to aid in standardizing the diagnostic criteria [7]. Using a combination of simple history and physical exam items, the CTS-6 was intended to be used by non-expert clinicians who frequently encounter hand numbness as a chief complaint [7]. Since its publication, the CTS-6 has been used as a reference standard to compare other diagnostic modalities and has demonstrated substantial levels of inter-rater reliability for the examination components [8]. The physical exam components of the CTS-6 include the presence of thenar atrophy/weakness, Phalen's test, static two-point discrimination (2PD) test, and a Tinel's sign [7]. Scoring within the CTS-6 is weighted by examination performance and was designed to be used in aggregate, as no single examination finding, in isolation, is diagnostic of CTS [9].

Although the CTS-6 has been established as a practical diagnostic tool, other physical exam maneuvers that are not included in the CTS-6 are often still utilized by physicians as part of the evaluation of peripheral compressive neuropathy. Carpal tunnel compression was described by Durkan in 1991 with an Sn/Sp of 0.87 and 0.90, respectively [10]. Subsequent publications have demonstrated wide variations in the Sn/Sp for the Durkan test with different examiners and methodologies [10-14]. Similarly, there has been interest in the scratch-collapse test for the diagnosis of CTS, with the initial series demonstrating a Sn of 0.64 [15]. However, subsequent blinded follow-up studies have reported a Sn of 0.24 and a Sp of 0.60, again demonstrating wide variation in exam validity [15-17]. Variability in the validity of these physical exam maneuvers introduces potential uncertainties in the diagnostic work-up of CTS. Considering the variability in examination performance characteristics for individual studies, aggregate data may more accurately reflect individual examination validity.

The purpose of this meta-analysis was to analyze the validity (Sn/Sp) of common physical examination tests and maneuvers utilized in the evaluation and diagnosis of CTS. We hypothesized that the reported Sn/Sp values for CTS physical examination maneuvers would be variable throughout the existing literature. 

## Review

Materials and methods 

This review follows the transparent reporting guidelines outlined in the Preferred Reporting Items for Systematic Reviews and Meta-Analyses (PRISMA) checklist. 

Literature Search

On March 24, 2022, a search of the MEDLINE (Medical Literature Analysis and Retrieval System Online) and Embase (Excerpta Medica Database) databases was done for English language literature on diagnostic physical exam maneuvers for CTS. Search strategies are detailed in Appendix 1. A date range was not set in order to capture as much published data as possible. All article formats were included in the index search. Non-human research, cadaveric studies, articles not available in English, and surgical techniques not related to physical exam tests used in the diagnosis of CTS were excluded. 

Study Selection and Data Extraction

Titles and citations were screened for duplicates. The remaining titles and abstracts were screened for eligibility independently by two authors (YO, DSH) with any discrepancy being resolved through discussion and consultation with a third author. Eligible full-text articles were assessed against the inclusion and exclusion criteria and data extracted. Data were extracted from studies including article name, total number of subjects/hands, type of exam, and examination diagnostic validity (Sn/Sp). 

Statistical Methods and Analysis

The overall relationship between Sn/Sp was displayed using scatterplots. For each individual examination, forest plots were presented to display the estimated results of Sn/Sp from the included studies. Boxplots were used to graphically demonstrate the locality, spread, and skewness of the Sn/Sp through the quartiles. Median and quartiles (Q1, Q3) were summarized for Sn/Sp, respectively. Statistical analyses were performed using RStudio (Posit Software, PBC, Boston, Massachusetts, United States). 

Results

**Figure 1:**
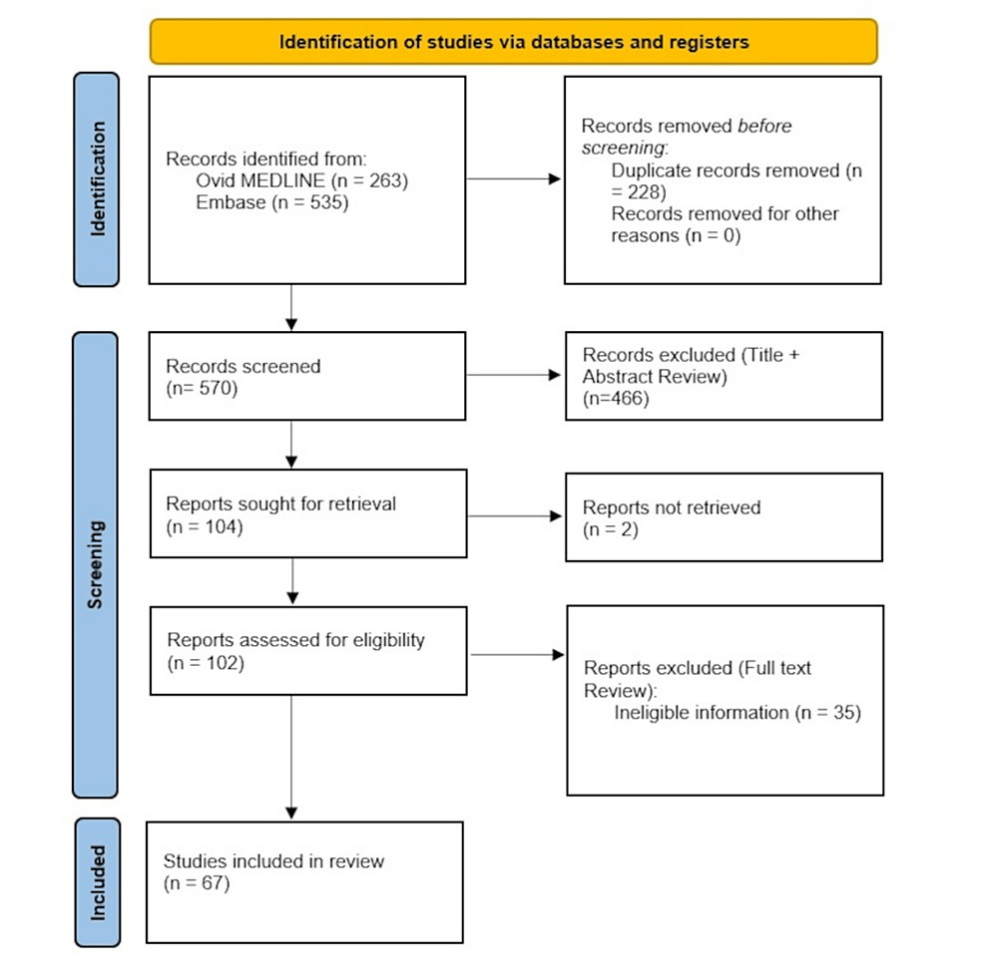
PRISMA flowchart for study selection PRISMA: Preferred Reporting Items for Systematic Reviews and Meta-Analyses

Figure 1 shows a flowchart of article inclusion through the study period. After initially screening 570 articles, 67 articles involving a total of 8722 patients (8924 hands) were finally included in the review. 

Basic demographic information of patients from the included articles and their bibliographic data are presented in Table 1. Of the included articles, 48 assessed Phalen's test, 45 assessed Tinel's sign, 21 assessed the Durkan test, seven assessed the scratch-collapse test, 11 assessed SWM, and six assessed 2PD as part of the diagnosis of CTS. 

**Table 1:** Basic demographic information of patients and bibliographic information of studies included in this meta-analysis. a: Gender was reported in only 51 articles. Age was reported for 56 articles.  Racial demographics were reported in a single article.

VARIABLE	VALUE
Total number of patients	8722
Total number of hands	8924
Male sex, n(%)^a^	2332 (27%)
Mean age in years (SD)^a^	44 (7.9)
Race (n)^a^	
White	77 (88%)
Black	5 (5%)
Asian	3 (3%)
Other	2 (2%)
Study design (n,%)	
Case Series	50 (74.5%)
Case-Control	16 (24%)
Randomized Controlled Trial	1 (1.5%)
Level of Evidence	
I	1 (1.5%)
II	0 (0%)
III	25 (37.3%)
IV	41 (61%)

**Figure 2:**
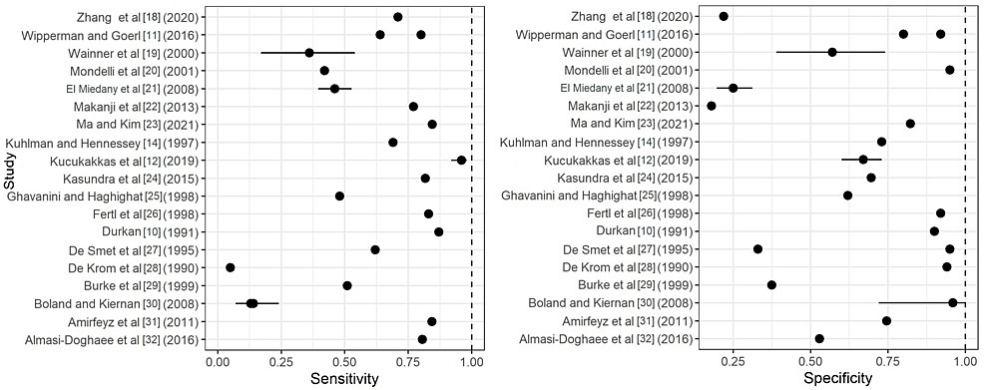
Forest plots of the estimated sensitivity (Sn) and specificity (Sp) for the Durkan test. Included articles: Zhang et al. [18], Wipperman and Goerl [11], Wainner et al. [19], Modelli et al. [20], El Miedany et al. [21], Makanji et al. [22], Ma and Kim [23], Kuhlman and Hennessey [14], Kucukakkas and Yurdakul, [12], Kasundra et al. [24], Ghavanini and Haghighat [25], Fertl et al. [26], Durkan [10], De Smet et al. [27], De Krom et al. [28], Burke et al. [29], Boland and Kiernan [30], Amirfeyz et al [31], Almasi-Doghaee et al. [32]

**Figure 3:**
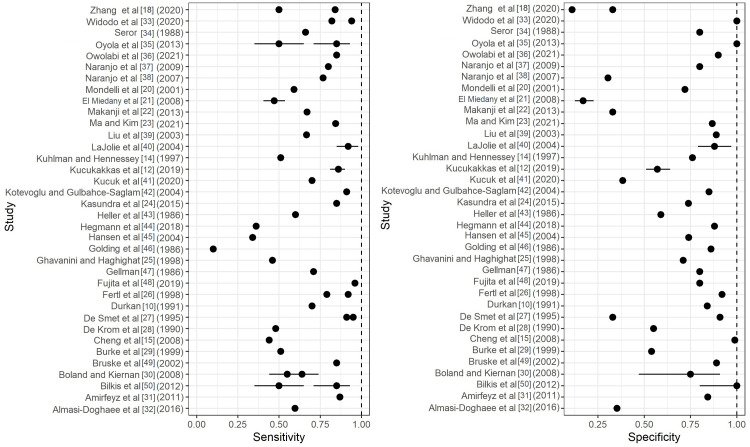
Forest plots of the estimated sensitivity (Sn) and specificity (Sp) for Phalen's test. Included articles: Zhang et al [18], Widodo et al. [33], Seror [34], Oyola et al. [35], Owolabi et al. [36], Naranjo et al. [37,38], Mondelli et al. [20], El Miedany etl al. [21], Makanji et al. [22], Ma and Kim [23], Liu et al. [39], LaJolie et al. [40], Kuhlman and Hennessey [14], Küçükakkaş and Yurdakul, [12], Kucuk et al. [41], Kotevoglu and Gulbahce-Saglam [42], Kasundra et al. [24], Heller et al. [43], Hegmann et al [44], Hansen et al. [45], Golding et al. [46], Ghavanini and Haghighat [25], Gellman [47], Fujita et al. [48], Fertl et al. [26], Durkan [10], De Smet et al. [27], De Krom et al. [28], Cheng et al. [15], Burke et al. [29], Bruske et al [49] Boland Kiernan [30], Bilkis et al. [50], Amirfeyz et al. [31], Almasi-Doghaee et al. [32]

**Figure 4:**
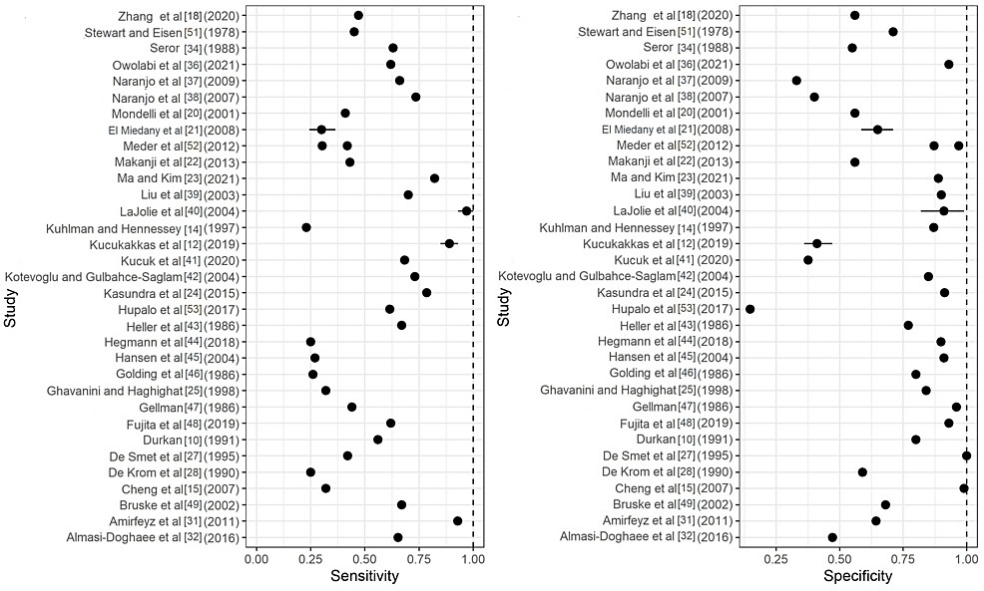
Forest plots of the estimated sensitivity (Sn) and specificity (Sp) for Tinel's test. Included articles: Zhang et al [18], Stewart and Eisen [51], Seror [34], Owolabi et al. [36], Naranjo et al. [37,38], Mondelli et al. [20], El Miedany etl al. [21], Meder et al. [52], Makanji et al. [22], Ma and Kim [23], Liu et al. [39], LaJolie et al. [40], Kuhlman and Hennessey [14], Küçükakkaş and Yurdakul [12], Kucuk et al. [41], Kotevoglu and Gulbahce-Saglam [42], Kasundra et al. [24], Hupalo et al. [53], Heller et al. [43], Hegmann et al [44], Hansen et al. [45], Golding et al. [46], Ghavanini and Haghighat [25], Gellman [47], Fujita et al. [48], , Durkan [10], De Smet et al. [27], De Krom et al. [28], Cheng et al. [15], Bruske et al. [49], Amirfeyz et al. [31], Almasi-Doghaee et al. [32]

Figures 2-4 depict forest plots of the estimated Sn/Sp for the Durkan test, Phalen's test, and Tinel's tests. Additionally, for each of the top six exam types, median and quartiles (Q1, Q3) were summarized for Sn/Sp (Table 2).

**Table 2:** Median and interquartile range (IQR) of sensitivity and specificity for each exam. SWM: Semmes-Weinstein monofilament; 2PD: static 2-point discrimination

	Included studies (n)	Sensitivity, Median (Q1, Q3)	Specificity, Median (Q1, Q3)
Phalen	48	0.70 (0.51, 0.85)	0.80 (0.58, 0.90)
Tinel	45	0.59 (0.32, 0.68)	0.80 (0.56, 0.91)
Durkan	21	0.67 (0.46, 0.82)	0.74 (0.53, 0.92)
Scratch-collapse	7	0.34 (0.22, 0.56)	0.78 (0.68, 0.93)
SWM	11	0.39 (0.11, 0.53)	0.85 (0.51, 0.89)
2PD	6	0.51 (0.33, 0.55)	0.90 (0.88, 0.90)

Sensitivity

The Sn of a test measures the ability to correctly diagnose CTS (also known as true positive rate). A higher Sn indicates fewer false negatives for an individual test. Phalen's test demonstrated the highest median Sn (0.70, (Q1, Q3): (0.51, 0.85)), followed by the Durkan test (0.67, (Q1, Q3): (0.46, 0.82)) as noted in Table 2.

Specificity

The Sp of a test measures the ability to correctly identify non-CTS cases (also known as true negative rate). A higher Sp indicates fewer false positives for an individual test. 2PD demonstrated the highest median Sp (0.90, (Q1, Q3): (0.88, 0.90)), followed by SWM (median = 0.85, (Q1, Q3): (0.51, 0.89)), as noted in Table 2.

**Figure 5:**
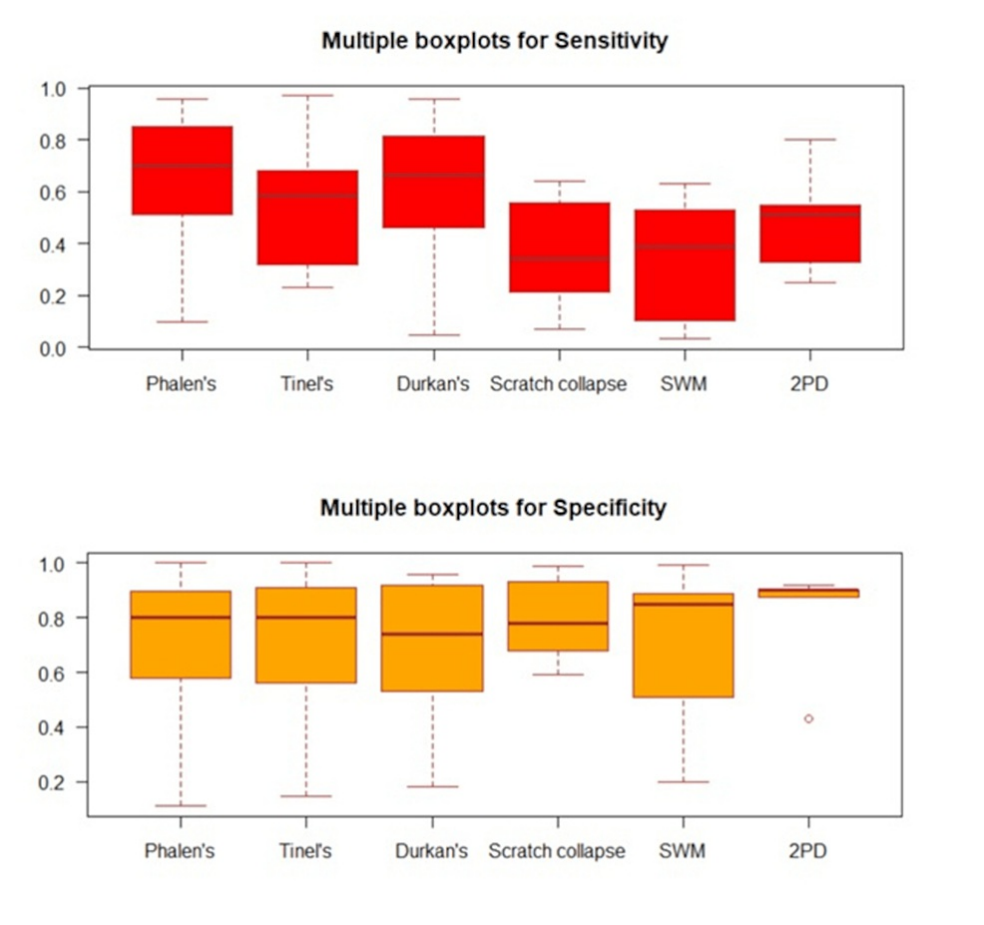
Multiple boxplots for the sensitivity (Sn) and specificity (Sp) values for the physical examination tests and maneuvers. SWM: Semmes-Weinstein monofilament; 2PD: static 2-point discrimination

Figure 5 depicts multiple boxplots showing the Sn/Sp of the six exam maneuvers. Table 3 contains a description of the study characteristics stratified by examination. 

**Table 3:** Study characteristics of included articles, stratified by exam type. SWM: Semmes-Weinstein monofilament; 2PD: static 2-point discrimination

EXAMINATION	Included Articles (n)	Total number of subjects (n)	Sample size, Median (Q1, Q3)	Prevalence of CTS (%), Median (Q1, Q3)	Age, Median (Q1, Q3)
Phalen	48	8,390	80 (42, 148)	73 (54, 90)	48 (45, 52)
Tinel	45	8,327	81 (42, 148)	74 (52, 90)	47 (44, 52)
Durkan	21	1,993	54 (42, 116)	84 (59, 97)	47 (45, 55)
Scratch-collapse	7	470	65 (40, 91)	72 (61, 75)	55 (53, 58)
SWM	11	2,730	110 (63, 368)	62 (47, 97)	47 (42, 47)
2PD	6	599	70 (60, 83)	82 (81, 97)	52 (49, 55)

Discussion

This meta-analysis investigated the reported diagnostic validity (Sn/Sp) for physical exam maneuvers used in the diagnosis and evaluation of CTS. These data indicate that prior publications have demonstrated high variability with respect to the performance of physical exam tests used in the evaluation of CTS. For examination maneuvers comprising portions of CTS-6, our results indicate highly variable examination performance with respect to Sn/Sp. The cumulative analysis showed that Phalen's test had a median Sn/Sp of 0.70/0.80. Tinel's sign and 2PD had a median Sn/Sp of 0.59/0.80 and 0.51/0.90, respectively. Published studies reveal there is controversy regarding which physical exam maneuver has the best performance characteristics within the CTS-6. For example, Zhang et al. reported Tinel's sign to have a higher Sp compared to Phalen's test [18]. However, other studies have reported the contrary, with Phalen’s test having higher Sn/Sp compared to Tinel’s sign [54,55]. Differences in reported Sn/Sp in individual exams within the CTS-6 are likely attributable to the study heterogeneity of included articles. Differences with respect to the prevalence of CTS, age, reference standard, and study methodology as well as cultural differences in patient populations, variable presentations, and the difference in compression may all contribute to the observed performance variability [40]. In this context, aggregate data may be more generalizable and more accurately reflect overall exam validity.

Phalen's test and 2PD had the highest Sn/Sp respectively in our meta-analysis and both studies are heavily weighted components of the CTS-6. With higher Sp, both Phalen's test and 2PD bolster the probability of the diagnosis of CTS when they are positive. Overall, points allocated within the CTS-6 are supported by our results. Phalen's test had the highest combined Sn/Sp across the literature and is allocated 5 points within the CTS-6, which is the highest for any single item. This compares to the 4.5 and 4 points allocated for 2PD and Tinel's sign, respectively, which both have high Sp (≥0.80), but lower Sn relative to Phalen's test.

Other examination maneuvers that are not used as part of the CTS-6 were additionally assessed in this investigation. When initially described, the scratch-collapse test was reported to have Sn/Sp of 0.64/0.69 for diagnosing CTS [15]. Blinded follow-up studies after this initial publication have described lower Sn/Sp values of 0.20/0.60 for the test [15,16]. Further studies on the validity of the scratch-collapse test have also reported similar outcomes. Cebron and Curtin reported a range of 0.24-0.77 for the Sn of the scratch collapse test for diagnosing CTS [56].

Areson et al. redemonstrated the low Sn/Sp of 0.48/0.59 for the scratch-collapse test and advised against the use of the test for clinical decision-making in the setting of CTS [57]. Overall, we found the reported Sn varied substantially between papers, ranging from 0.05 to 0.64. Again, this variability is likely due to heterogeneous methodologies implemented across the included studies. Considering the scratch-collapse test’s median Sn of 0.34, our meta-analysis is consistent with recent literature regarding the low validity of the scratch-collapse test. In this context, aggregate analyses, such as systematic reviews or meta-analyses, may provide a more balanced assessment of examination performance and test validity.

The variable performance of physical examination maneuvers noted in our series is also reflected in prior publications assessing imaging and advanced studies utilized in the work-up of CTS. Fowler et al., in their meta-analysis, showed that the Sn for diagnostic ultrasound ranged between 0.57 and 0.98 and the Sp ranged from 0.63 to 1.00 [58]. Landau et al. reviewed the use of power Doppler ultrasound and found that Sn/Sp for diagnosing CTS showed high levels of variability, reporting a range of 0.02-0.93 for Sn and an Sp of 0.89-1.00 [59]. The lack of an accepted diagnostic gold standard or reference standard for CTS likely contributes to this diagnostic uncertainty and serves as a caution against using individual tests in isolation to make the diagnosis.

This meta-analysis has a number of limitations, including the heterogeneity of the study types included. We did not limit our inclusion criteria by study type, including RCTs, case series, and case-control studies. Not all studies that were analyzed used the same reference standard to establish the diagnosis of CTS. The majority of studies in this meta-analysis used EDS as their reference standard, while the remaining studies used a variety of different reference standards such as clinical examinations (CTS-6), imaging studies (sonography), or a combination of these diagnostic techniques. Several different patient populations such as military members, dental workers, sex-specific studies, patients with rheumatoid arthritis, and hospitalized patients have been included in the studies assessed in our meta-analysis [23,27,34,60]. These heterogeneous populations can introduce potential confounding by the presence of selection and Berksonian biases [23,27,34,39,60]. 

## Conclusions

Our meta-analysis identified 67 publications that have reported Sn/Sp values for physical examination tests and maneuvers utilized in the evaluation of CTS. The results indicate considerable variability in the Sn/Sp of physical exam tests used in the diagnosis of CTS reported across individual studies. Phalen's test demonstrated the highest Sn as well as combined Sn/Sp, whereas 2PD demonstrated the highest Sp. Both of these examinations are incorporated in the CTS-6 with proportionally higher diagnostic weighting. Clinicians should be aware of the inherent limitations of each diagnostic test and their individual performance characteristics in aggregate, as opposed to the values reported in a single study. In the absence of a uniformly accepted diagnostic gold standard, a combination of exams, along with pertinent patient history, should guide the diagnosis of CTS.

## Appendices

 Appendix 1

**Table 4:** Search strategies and terms [mp=title, book title, abstract, original title, name of substance word, subject heading word, floating sub-heading word, keyword heading word, organism supplementary concept word, protocol supplementary concept word, rare disease supplementary concept word, unique identifier, synonyms]

EMBASE & MEDLINE Search Terms and Strategies
1. carpal tunnel syndrome.mp
2. physical exam.mp
3. carpal tunnel.mp
4. carpal.mp
5. tunnel.mp
6. median nerve.mp
7. Tinel.mp
8. scratch collapse.mp
9. Durkan.mp
10. Phalen.mp
11. thenar atrophy.mp
12. two point discrimination.mp
13. 1 and 2
14. 2 and 3
15. 2 and 4 and 5
16. 2 and 6
17. 1 and 3 and 7
18. 1 and 3 and 8
19. 1 and 3 and 9
20. 1 and 3 and 10
21. 1 and 3 and 11
22. 1 and 3 and 12
23. 13 or 14 or 15 or 16 or 17 or 17 or 19 or 20 or 21 or 22
24. limit 23 to English language
25. limit 24 to human
26. 24 and 25
